# Comparison of nuisance parameters in pediatric versus adult randomized trials: a meta-epidemiologic empirical evaluation

**DOI:** 10.1186/s12874-017-0456-8

**Published:** 2018-01-10

**Authors:** Ben Vandermeer, Ingeborg van der Tweel, Marijke C. Jansen-van der Weide, Stephanie S. Weinreich, Despina G. Contopoulos-Ioannidis, Dirk Bassler, Ricardo M. Fernandes, Lisa Askie, Haroon Saloojee, Paola Baiardi, Susan S. Ellenberg, Johanna H. van der Lee

**Affiliations:** 1 Department of Pediatrics, Alberta Research Centre for Health Evidence, University of Alberta, Edmonton, Canada; 2 Department of Biostatistics, Julius Centre for Health Sciences and Primary Care, University Medical Centre, Utrecht, Netherlands; 3 Pediatric Clinical Research Office, Emma Children’s Hospital, Academic Medical Centre, Amsterdam, Netherlands; 4 Department of Clinical Genetics, Amsterdam Public Health research institute, VU University Medical Center, Amsterdam, The Netherlands; 5 Department of Pediatrics, Division of Infectious Diseases, Stanford University School of Medicine, Stanford, CA USA; 6 Meta-Research Innovation Center at Stanford (METRICS), Stanford University, Stanford, CA USA; 7 Department of Neonatology, University Hospital, Zurich and University of Zurich, Zurich, Switzerland; 8 Clinical Pharmacology and Therapeutics Unit, Faculty of Medicine, Instituto de Medicina Molecular, University of Lisbon, Lisbon, Portugal; 9 Department of Pediatrics, Santa Maria Hospital, Lisbon, Portugal; 10 NHMRC Clinical Trials Centre, University of Sydney, Sydney, Australia; 11 Division of Community Pediatrics, Department of Pediatrics and Child Health, University of the Witwatersrand, Johannesburg, South Africa; 12 Salvatore Maugeri Foundation, Pavia, Italy; 13 Department of Biostatistics and Epidemiology, University of Pennsylvania, Perelman School of Medicine, Philadelphia, PA USA

**Keywords:** Nuisance parameters, Extrapolation, Sample size computations, Pediatric trials, Adult trials

## Abstract

**Background:**

We wished to compare the nuisance parameters of pediatric vs. adult randomized-trials (RCTs) and determine if the latter can be used in sample size computations of the former.

**Methods:**

In this meta-epidemiologic empirical evaluation we examined meta-analyses from the Cochrane Database of Systematic-Reviews, with at least one pediatric-RCT and at least one adult-RCT. Within each meta-analysis of binary efficacy-outcomes, we calculated the pooled-control-group event-rate (CER) across separately all pediatric and adult-trials, using random-effect models and subsequently calculated the control-group event-rate risk-ratio (CER-RR) of the pooled-pediatric-CERs vs. adult-CERs. Within each meta-analysis with continuous outcomes we calculated the pooled-control-group effect standard deviation (CE-SD) across separately all pediatric and adult-trials and subsequently calculated the CE-SD-ratio of the pooled-pediatric-CE-SDs vs. adult-CE-SDs. We then calculated across all meta-analyses the pooled-CER-RRs and pooled-CE-SD-ratios (primary endpoints) and the pooled-magnitude of effect-sizes of CER-RRs and CE-SD-ratios using REMs. A ratio < 1 indicates that pediatric trials have smaller nuisance parameters than adult trials.

**Results:**

We analyzed 208 meta-analyses (135 for binary-outcomes, 73 for continuous-outcomes). For binary outcomes, pediatric-RCTs had on average 10% smaller CERs than adult-RCTs (summary-CE-RR: 0.90; 95% CI: 0.83, 0.98). For mortality outcomes the summary-CE-RR was 0.48 (95% CIs: 0.31, 0.74). For continuous outcomes, pediatric-RCTs had on average 26% smaller CE-SDs than adult-RCTs (summary-CE-SD-ratio: 0.74).

**Conclusions:**

Clinically relevant differences in nuisance parameters between pediatric and adult trials were detected. These differences have implications for design of future studies. Extrapolation of nuisance parameters for sample-sizes calculations from adult-trials to pediatric-trials should be cautiously done.

## Background

For sample size calculations for randomized controlled trials (RCTs), some parameters, like the treatment difference (effect size) between the experimental and control intervention have to be estimated. The investigators also have to estimate parameters that are not of direct interest, but are required for the computations. These parameters are often termed *nuisance parameters*, the most common of which are the control-group event rate (CER) for binary outcomes and the standard deviation (SD) for continuous outcomes.

Determining an appropriate sample size for an RCT has always been a challenge due to these nuisance parameters being unknown and needing to be estimated [1, 2]. Usually, they can only be estimated from previous studies on the same topic, but there is always a problem when the new study is the first trial on the topic. Consequences of erroneously estimated nuisance parameters are the under-estimation or over-estimation of the required sample size, which can then lead to underpowered studies that may fail to reach a definitive conclusion [3] in the former case, or to unnecessary higher study cost and longer recruitment periods in the latter case [4].

Investigators sometimes extrapolate evidence on nuisance parameters from randomized trials in adults for sample size calculations in pediatric trials [1, 5, 6]. However, differences in clinical effects between adults and children do exist as has been previously shown by systematic empirical evaluations of the comparative effectiveness and comparative safety of medical interventions between adults and children [7–10]. A systematic empirical evaluation of nuisance parameters in pediatric RCTs, as compared to adult RCTs has not been previously performed.

We performed a meta-epidemiologic empirical evaluation to investigate whether nuisance parameters differ between pediatric and adult trials on the same topics, for the same compared interventions and for the same clinical outcomes. We also studied whether there are differences in the nuisance parameters according to the types of outcomes: binary versus continuous efficacy outcomes and mortality versus non-mortality outcomes.

## Methods

### Selection of meta-analyses

We addressed the above questions by examining 106 systematic reviews from the Cochrane Database of Systematic Reviews (CDSR) (Issue 1, 2007), that have been previously analyzed [9] in an empirical evaluation of the comparative effectiveness of medical interventions in children versus adults. These systematic reviews included 135 meta-analyses on diverse medical interventions with binary efficacy outcomes with at least one adult RCT and at least one pediatric RCT per meta-analysis. In this prior analysis, the following types of meta-analyses were excluded: those involving surgical, psychological, behavioral, social interventions, or evaluations of medical devices; those focusing exclusively on harms, without any primary efficacy outcome; meta-analyses with only continuous outcomes; meta-analyses for which it was not possible to discriminate between an experimental and control intervention; those without any quantitative data synthesis and those that did not cover both age groups and did not have any complementary systematic review focusing on the other age group. When a systematic review addressed different types of eligible comparisons of experimental versus control interventions, each comparison was considered for eligibility separately [9].

**Fig. 1:**
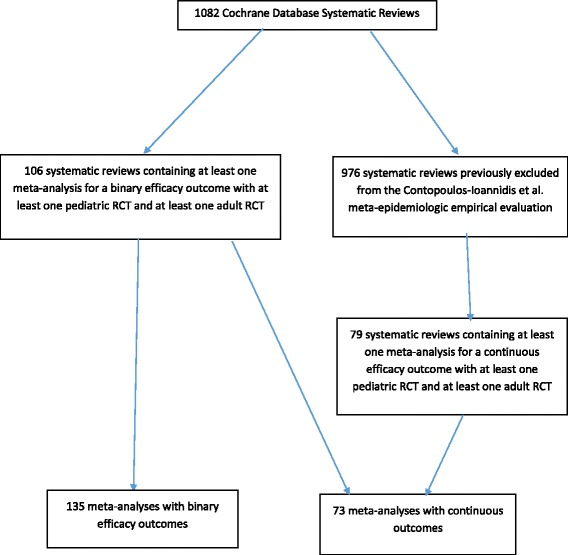
Flowchart

We further screened these 106 systematic reviews to identify additional eligible meta-analyses with continuous outcomes that had included at least one adult RCT and at least one pediatric RCT per meta-analysis. Furthermore, we screened 79 reviews previously excluded in the Contopoulos-Ioannidis et al. study since they did not contain binary efficacy primary outcomes. Four authors (BV, IT, MJW, and SW) extracted all continuous-outcome meta-analyses up to a maximum of five per review—if more than five continuous-outcome meta-analyses were reported we chose the five meta-analyses that had the maximum number of participants. We excluded meta-analyses that used a standardized mean difference as their method of pooling, since these standard deviations would be expected to be different (i.e. different scales for different trials). A total of 73 continuous-outcome meta-analyses (from both sources) were included in our continuous-outcome analysis (Fig. 1).

### Data extraction

From each eligible meta-analysis with binary efficacy outcomes we extracted the following data from the included RCTs: a) compared interventions (experimental vs. control); b) event rate (events/total) in the control group; c) control group sample size and d) age group of study participants (adults versus children). From each eligible meta-analysis with continuous outcomes we extracted the following study level data: a) standard deviation of effect-size in the control group; b) control group sample size; c) mean of effect size in the control group and d) age group of study participants. For the identification of the experimental and control intervention when two active interventions were compared we used the interpretation of the authors of the Cochrane review. For the study age group categorization, we used the classification reported in the Cochrane review. If this was not described, we used age group classification rules previously applied by Contopoulos-Ioannidis et al. In brief, a study was characterized as “adult” if all included patients were >12 years and patients >20 years were also included; “pediatric” if all patients were <20 years and patients <12 years were also included.

### Data synthesis

### Primary endpoints

#### Binary efficacy outcomes

*Pooling of CER- risk ratios (summary-CER-RRs)*: First, we calculated the CER by dividing the event rate in the control group by the total sample size in the control group for each individual study. Second, for each of these CERs we calculated its standard error using the normal scores method [11]. Third, separately for all pediatric and adult RCTs, we used a random effects model [12] within each meta-analysis to estimate a pooled-pediatric-CER and pooled-adult-CER respectively, its standard error and the heterogeneity statistic *I-squared* [13]. Fourth, for each meta-analysis, we computed the logarithm of the CER-RR of the pediatric CER to the adult CER with its associated standard error. Finally, we calculated the summary-CER-RR between pediatric and adult trials and their 95% confidence intervals across all meta-analyses by synthesizing the pooled logarithms of the CER-RRs within each meta-analysis again using the random effects model [14]. The logarithms were converted back to CER-RRs for presentation purposes. A CER-RR < 1 indicated that pediatric trials had smaller CERs than adult trials.

#### Exploratory analyses

*Pooling of magnitude of absolute CER-RRs*: Here our interest was in the magnitude of difference in estimated nuisance parameters, not the direction. The pooling of the CERs as described above takes into account also directional differences in CER-RRs between pediatric and adult studies—thus, a minimal estimated difference in that analysis would not reduce concerns about the comparability of nuisance parameters in any individual situation. Therefore, it was important to assess also the magnitude of the differences by performing a separate meta-analysis of the “absolute CER-RRs”. The methodology was the same as above, except that before the final step of pooling the CER-RRs, we replaced any RR smaller than 1 with its reciprocal. This is mathematically equivalent to replacing the logarithm of the RR in the final meta-meta-analysis with its absolute value. This pooled estimate tells us how large (on average) the CER-RRs were, regardless of which group (pediatric or adult) had the larger CER. For example, CER-RRs of 1.25 and 0.80 will be transformed to absolute CER-RRs of 1.25 and 1.25 respectively.

### Subgroup analysis

The binary-outcome meta-analyses were sub-grouped by mortality and non-mortality outcomes. We computed a separate summary--CER-RR for each of these groups.

#### Continuous efficacy outcomes

*Pooling of control group-effect-SD-ratios (CE-SD-ratios)*: First, we extracted the SD of the estimate in the control group of each pediatric and adult RCT. Second, we calculated the weighted average of the SDs for all pediatric (pooled-pediatric-CE-SD) and adult RCTs (pooled-adult-CE-SD), respectively, within each meta-analysis, by weighting with the square root of each study’s sample size. Third, we computed the ratio [CE-SD-ratio] of the pediatric vs. adult control-group effect-SDs within each meta-analysis by dividing the weighted-average-pediatric SDs (pooled-pediatric-CE-SD) by the weighted-average-adult SDs (pooled-adult-CE-SD). Finally, we calculated the summary-CE-SD-ratio between pediatric and adult trials across all meta-analyses as the weighted average of the logarithms of these ratios. The summary log SD ratio was exponentiated to get the summary CE-SD-ratio. A CE-SD-ratio < 1 indicates that pediatric trials had smaller SDs than adult trials.

### Descriptive analyses

For graphical comparison of the CE-SDs of adult vs. pediatric RCTs within each meta-analysis we divided the CE-SDs of each individual RCT by the maximum SD in that meta-analysis in order to get a standardized CE-SD for each adult and pediatric RCT that would allow comparisons of adult and pediatric CE-SDs within each meta-analysis and across meta-analyses.

#### Software

Summary statistics and weighted averages were computed in SAS 9.3 (SAS institute Inc., Cary NC). Meta-analyses were performed using the *metan* modul*e* in Stata 11.2 (StataCorp, College Station TX). Graphs were produced using Review Manager, SPlus 8.2 (Tibco Software Inc.), and Microsoft Excel.

## Results

We examined 208 meta-analyses, 135 with binary primary efficacy outcome data and 73 with continuous outcome data, from a total of 185 systematic reviews. All meta-analyses comprised a total of 2110 RCTs; 1515 adult RCTs (1126 with binary and 389 with continuous outcomes) and 595 pediatric RCTs (355 with binary and 240 with continuous outcomes). Each study could have contributed data to more than one meta-analysis within each systematic review (e.g. for different meta-analyses with different compared interventions or outcomes).

### Binary outcome analyses

#### Summary pooled-CER-RRs

**Fig. 2:**
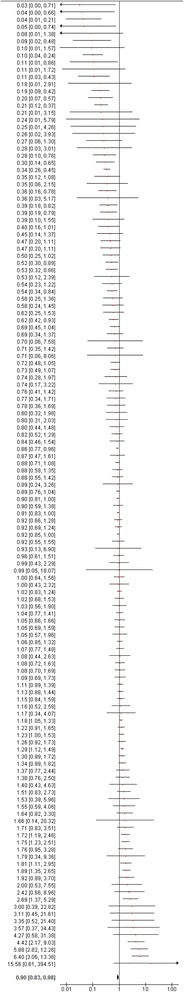
Distribution across all meta-analyses of the CER-Risk Ratios of the logarithmically transformed pooled-pediatric-CERs vs. pooled-adult-CERs per meta-analysis

**Fig. 3:**
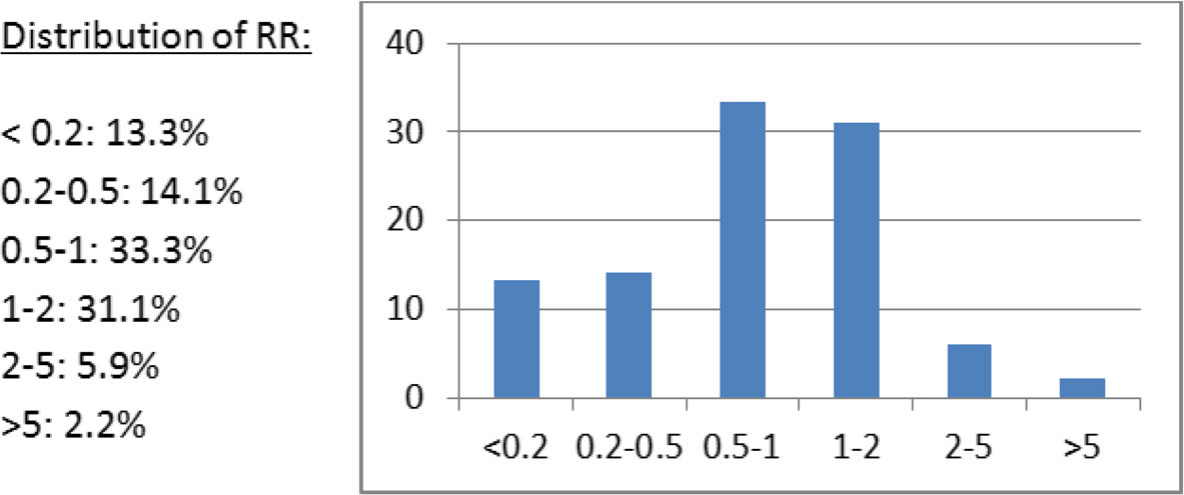
Distribution of CER-RR within each meta-analysis

The summary-pooled-CER-RR of pediatric CERs versus adult CERs across all 135 meta-analyses showed that pediatric RCTs had on average a 10% smaller CERs (summary-CER-RR: 0.90; 95% CI: 0.83, 0.98). The individual CER-RRs within each meta-analysis are shown in Fig. 2 and their distribution thereof in Fig. 3. Overall, 13.3% of the examined meta-analyses had pediatric-CERs that were at least five-fold smaller than the adult-CERs; the opposite (adult RCTs with at least five-fold smaller CERs than pediatric RCTs) occurred in only 2.2% of cases. Moreover, in 60.7% of the meta-analyses, the pediatric-CERs were smaller than the adult-CERs.

*I*^*2*^ values were calculated for 172 of the 270 meta-analyses (135 each for pediatric and adult trials respectively); for the remaining meta-analyses, *I*^*2*^ was not defined as they included only single studies. The distribution of these *I*^*2*^ values within each meta-analysis is shown in Table 1. Since these meta-analyses do not mix pediatric and adult data, these results show high heterogeneity among studies even when the pediatric/adult factor was taken out of the analysis.

**Table 1:** Distribution of I^2^ values across meta-analyses

I-squared	Number of meta-analyses (%)
0%	30 (17%)
0–20	7 (4%)
20–40	5 (3%)
40–60	15 (9%)
60–80	32 (19%)
80–100	83 (48%)

#### Subgroup analysis: Mortality and non-mortality outcomes

There were 21 meta-analyses with mortality outcomes and 114 meta-analyses with non-mortality outcomes. Among the 21 meta-analyses with mortality outcomes the summary-pooled CER-RRs of pediatric-CERs vs. adult-CERs was 0.48 (95% CI: 0.31, 0.74). In 18 of these 21 meta-analyses the mortality CER in adult trials was larger than that in pediatric trials.

Among the 114 meta-analyses with non-mortality outcomes the summary-CER-RR of pediatric vs. adult trials was 1.00 (95% CI: 0.92, 1.09).

#### Pooling using magnitude (absolute CER-RR)

**Fig. 4:**
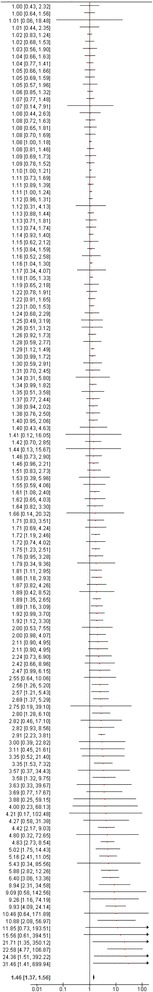
Distribution of the absolute values of the CER-Risk Ratios of the logarithmically transformed pooled-pediatric-CERs vs. pooled-adult CERs per meta-analysis

When we repeated the primary analysis using the “absolute-CER-RR” (Fig. 4) instead of the CER-RR the summary--absolute-CER-RR was 1.46 (95% CI; 1.37, 1.56). This indicates that CERs in pediatric trials are on average either 1.5 times larger or 1.5 times smaller than in adult trials.

### Continuous outcome analyses

#### Summary-pooled-CE-SD-ratios

**Fig. 5:**
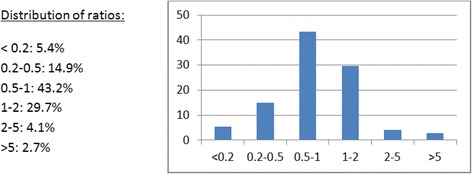
Summary of CE-SD ratios of pooled-pediatric-CE-SDs vs. pooled-adult-CE-SDs (within each meta-analysis)

The weighted average (weighted on the log scale by the square root of the sample size) (summary-CE-SD-ratio) of the CE-SD-ratios of pediatric-CE-SDs vs adult-CE-SDs for the 73 meta-analyses with continuous efficacy outcomes was 0.74. This indicates that on average the pediatric RCTs CE-SDs were 26% smaller than their adult counterparts. The distribution of the CE-SD-ratios is shown in Fig. 5. In 27.1% of the meta-analyses the CE-SD-ratios between pediatric and adult RCTs differed by at least 2-folds in either direction. Furthermore, ignoring direction of difference, and looking only at the sizes, the weighted magnitude of CE-SD-ratio was 1.76, which means that, on average, the CE-SD in adult and pediatric RCTs differed by a factor of 1.76.

**Fig. 6:**
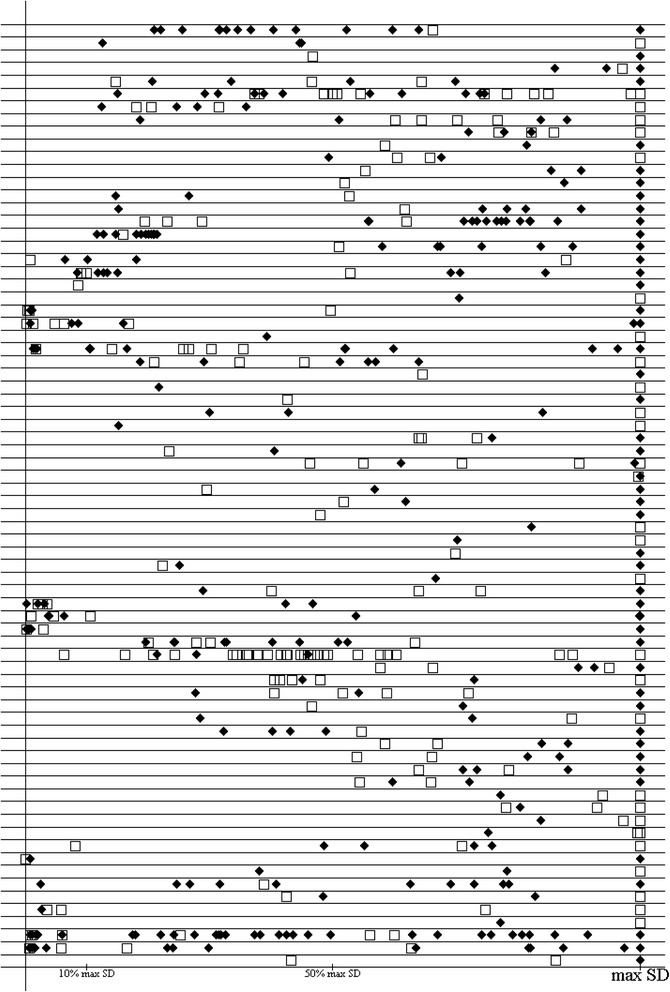
Standardized control-group-effect SDs (CE-SDs) of pediatric and adult trials within each meta-analysis for meta-analyses with continuous outcomes. Each horizontal bar represents one meta-analysis; white square = pediatric trial; black diamond = adult trial; max SD = the SD of the meta-analysis’s study with the maximum effect-size CE-SD. Reading graph: Example: the first row shows that in this meta-analysis there were fifteen studies: fourteen adult studies and one pediatric study. The largest SD was an adult study. The other adult studies’ CE-SDs ranged from 22% to 64% of the value of the largest, while the lone pediatric study had an SD that was 66% that of the adult study

The distribution of the standardized SDs of the effect sizes in the control groups of all adult and pediatric studies within each meta-analysis is shown in Fig. 6. The SDs of the effect sizes in the control groups of pediatric vs. adult trials varied greatly both within meta-analyses and between meta-analyses.

## Discussion

The control-group event rate nuisance parameter in pediatric trials was on average 10% smaller than that in adult trials. In our secondary analyses, when we considered the magnitude of the control-group effect size rather than the direction of effect, pediatric trials had an average control-group event rate that was 1.5 times higher or 1.5 times smaller than that in adult RCTs. The reason for considering also the magnitude of effects, ignoring their direction thereof, is that an important issue to address is whether nuisance parameters in pediatric studies are likely to differ from those estimated from adult studies. We could have large differences in nuisance parameters (some over-estimating, some under-estimating) that average out to no difference. By analyzing magnitudes, we are pre-supposing a difference and trying to estimate how large that difference might be.

In over 60% of meta-analyses the control-group event rates in pediatric RCTs were smaller than those in adult trials and in 36% of the meta-analyses, relative differences in control-group event rates of at least 2-folds, in either direction, were identified. Specifically, for mortality outcomes, the control-group mortality rate in pediatric trials was on average 50% lower than that in adult trials.

Large variation was also seen between pediatric and adult trials when continuous efficacy outcomes were considered. The pediatric control-group SD was on average 26% smaller than that of adult trials and in 27% of the meta-analyses the relative difference in SDs between pediatric and adult trials was at least 2-fold in either direction. Moreover, when the magnitude of the control-group SD was considered, pediatric trial SDs were at least either 1.8 times larger or 1.8 times smaller than adult trial SDs.

Large differences were seen among many studies with regards to nuisance parameters. To demonstrate how erroneous estimation of nuisance parameters can affect sample size computation substantially, we will take two examples from the included meta-analyses in this study. In the review *Antibiotics for the common cold and acute purulent rhinitis*, for the primary meta-analysis of the persisting symptoms outcome, we had an estimated CER in the adult population of 0.48. If one wishes to conduct a pediatric trial on the same topic, with a type I error probability of 0.05, 80% power, and an assumption that a 30% reduction in number of patients with persisting symptoms would be required to demonstrate a clinically relevant antibiotic effect, the required sample size for the pediatric study would be 182 patients per arm. Under the assumption of a CER of 0.048, as was actually seen in the pediatric trials, the required sample size would be over 16 times larger at 2962. To give an example using a continuous outcome, we take the review *Early emergency department treatment of acute asthma with systemic corticosteroids*. For the outcome of final PEFR, we had observed a mean SD in the adult studies of 32 L/min. Suppose we plan to conduct a pediatric trial on the same topic, using a type I error probability of 0.05, a power of 80%, and a minimal clinical important difference threshold of 15 L/min. Using the adult estimated SD of 32 L/min, we could compute that we would require 643 patients in each of two groups. If we assume an SD estimate of 4.3 L/min, as was observed in the pediatric trials, we would only require a sample of 12 patients in each group, a sample size that is less than 2% of the originally computed sample size. It is clear from these examples that the erroneous estimations of these nuisance parameters can have important implications in the sample size computations, which can lead to either inappropriately powered studies that would not be able to answer the clinical question, or, on the other hand, to unnecessary waste of valuable clinical and financial resources. A third unwanted consequence might be that a proposed trial is not conducted because the erroneous estimate for the sample size is too large to be feasible.

We did observe a trend in both binary and continuous outcome data for pediatric RCTs to have smaller values of nuisance parameters (both CERs and CE-SDs) than their adult counterparts. Thus, when one does use these parameters from adult studies as surrogate for pediatric studies, the nuisance parameter is more likely to be overestimated than underestimated. This relationship has been well documented and graphed^2^. In the case of continuous data, an overestimation of the SD will always result in an overestimation of the sample size. The situation for binary data is more nuanced, as the sample size will depend upon the ratio of the CER and the treatment-group event rate (the closer the ratio is to 1, the larger the required sample size), so an underestimation of the CER could lead to either an under- or overestimated sample size. For example, if a pediatric population had an actual CER of 0.3 with a treatment-group event rate of 0.2, then underestimating the CER (say as 0.25) would result in a larger than required sample. However; if the treatment-group event rate was 0.4, then this underestimate of the CER would result in a sample that was too small.

The discrepancies in the nuisance parameters between pediatric and adult trials were more prominent with mortality outcomes. In 86% of meta-analyses with mortality outcomes, the mortality CER in adult trials was larger than that in pediatric trials. On average, the control-group mortality rates in adult trials were two times larger than in pediatric trials. Mortality seems to be an outcome where extrapolation of adult control-group event rates for the estimation of pediatric trial sample sizes may give inaccurate results.

We should acknowledge some study limitations. Traditional meta-analysis of standard deviations was not feasible in the analysis of continuous outcomes since the systematic reviews did not provide us enough information to ascertain variances around these nuisance parameters. Meta-analyses were done on a variety of outcomes, and thus the standard deviations were all reported in different units, and therefore not comparable across meta-analyses without standardization. In both the analyses of binary and continuous outcomes we observed considerable heterogeneity in nuisance parameters, not only between meta-analyses but also within them. We assumed that studies included within the same meta-analysis of a Cochrane review would have populations sufficiently similar to use them to impute nuisance parameters. However, extremely high between-study heterogeneity (I^2^ > 80%) was seen in more than half of the meta-analyses, which implies that even within studies of the same age-group (i.e. adult or pediatric) we cannot expect nuisance parameters to routinely be similar. This suggests that not only should we be wary of extrapolating nuisance parameters for pediatric studies from adult studies, but we should be almost equally wary of extrapolating them from other pediatric studies.

With these limitations in mind and given the results we have seen here, it would be interesting to do a further and more refined analysis as to which factors may lead to better concordance between the nuisance parameters of pediatric and adult studies. This would be a difficult endeavor, however, since these factors would likely be specific to a subject area, and not necessarily generalizable. Analysis would then have to be limited to those areas where there are enough studies to do it properly.

## Conclusion

This study provides evidence to raise awareness among investigators planning to design trials in children, when available data on nuisance parameters are mostly from adult studies that significant differences between pediatric and adult trials do exist. Extrapolation from adult trials of nuisance parameters to guide sample size calculations for pediatric trials should be cautiously done. Inappropriate extrapolations of nuisance parameters from adult trials to pediatric trials can lead to erroneous sample size calculations. Significant over- or underestimation of the required pediatric sample sizes can occur particularly when the outcome is mortality. When there is doubt about the similarity between the population from which the estimates are derived and the prospective study population, either a blinded sample size review during the early phase of the new trial (internal pilot study [15]), a more flexible (sequential) design and analysis [1, 2], or use of a standardized effect size [16] should be considered to maximize trial efficiency.

## Abbreviations

CDSR: Cochrane database of systematic reviews
CER: Control-group event rate
CER-RR: Control-group event rate risk ratio
CE-SD: Control-group effect standard deviation
RCT: Randomized control trial
SD: Standard deviation
